# Gene network-based analysis identifies two potential subtypes of small intestinal neuroendocrine tumors

**DOI:** 10.1186/1471-2164-15-595

**Published:** 2014-07-15

**Authors:** Mark Kidd, Irvin M Modlin, Ignat Drozdov

**Affiliations:** Yale University School of Medicine, New Haven, CT 06510 USA; Bering Limited, Richmond, UK

**Keywords:** Blood, Gene marker, Microarray, Network, Neurodevelopment, Neuroendocrine tumor, qPCR, Secretome, Transcript

## Abstract

**Background:**

Tumor transcriptomes contain information of critical value to understanding the different capacities of a cell at both a physiological and pathological level. In terms of clinical relevance, they provide information regarding the cellular “toolbox” e.g., pathways associated with malignancy and metastasis or drug dependency. Exploration of this resource can therefore be leveraged as a translational tool to better manage and assess neoplastic behavior. The availability of public genome-wide expression datasets, provide an opportunity to reassess neuroendocrine tumors at a more fundamental level. We hypothesized that stringent analysis of expression profiles as well as regulatory networks of the neoplastic cell would provide novel information that facilitates further delineation of the genomic basis of small intestinal neuroendocrine tumors.

**Results:**

We re-analyzed two publically available small intestinal tumor transcriptomes using stringent quality control parameters and network-based approaches and validated expression of core secretory regulatory elements e.g., CPE, PCSK1, secretogranins, including genes involved in depolarization e.g., SCN3A, as well as transcription factors associated with neurodevelopment (NKX2-2, NeuroD1, INSM1) and glucose homeostasis (APLP1). The candidate metastasis-associated transcription factor, ST18, was highly expressed (>14-fold, *p* < 0.004). Genes previously associated with neoplasia, CEBPA and SDHD, were decreased in expression (-1.5 – -2, *p* < 0.02). Genomic interrogation indicated that intestinal tumors may consist of two different subtypes, serotonin-producing neoplasms and serotonin/substance P/tachykinin lesions. QPCR validation in an independent dataset (*n* = 13 neuroendocrine tumors), confirmed up-regulated expression of 87% of genes (13/15).

**Conclusions:**

An integrated cellular transcriptomic analysis of small intestinal neuroendocrine tumors identified that they are regulated at a developmental level, have key activation of hypoxic pathways (a known regulator of malignant stem cell phenotypes) as well as activation of genes involved in apoptosis and proliferation. Further refinement of these analyses by RNAseq studies of large-scale databases will enable definition of individual master regulators and facilitate the development of novel tissue and blood-based tools to better understand diagnose and treat tumors.

**Electronic supplementary material:**

The online version of this article (doi:10.1186/1471-2164-15-595) contains supplementary material, which is available to authorized users.

## Background

Neuroendocrine neoplasms (NENs) or NETs represent 1-2% of all neoplasia and are comparable in incidence to testicular cancer, gliomas and Hodgkin’s lymphoma [1]. The most common variety, constituting approximately 29% of all NETs, develops within the small intestine or “midgut” and are the most common tumor of the small intestine [2, 3]. Although previously considered to be benign, they are indolent cancers (~60% overall five year survival rate) exhibiting a better survivals than adenocarcinomas of the same location [2, 4]. Although their biological behavior is generally non-aggressive, metastatic invasion is evident in 50% of tumors <1 cm [2]. The modest prognosis reflects the inherent clinical difficulty in diagnosis of small intestinal malignancy; disease may often have been present for some time before identification [2].

NETs are considered to be derived from neuroendocrine cells within the diffuse neuroendocrine system [5]. Like normal neuroendocrine cells, tumors exhibit a functional secretory apparatus e.g., chromogranins and proteins involved in amine uptake e.g., VMATs, as well as vesicular trafficking and fusions e.g., SNAP25 [6–9]. In addition, well-described signaling pathways involving G-protein coupled receptors such as somatostatin and dopamine have been defined e.g., cAMP/PKA [10, 11]. These have provided the basis for establishment of a histological classification, the development of targeted agents e.g., peptide receptor radiotherapy, as well as imaging strategies that utilize identification of cellular amine uptake mechanisms [12, 13]. The transcriptomic basis of tumor development and malignancy, however, remains largely unknown.

Chromosomal-based studies [14, 15] e.g., CGH and high resolution SNP arrays [16] and molecular profiling through exome analyses have identified alterations e.g., loss of 18q22-mer [17, 18] or SMAD4 LOH [19], that may be associated with neuroendocrine neoplasia. Similarly, gene expression profiling has identified a plethora of “marker genes” that include NAP1L1 [20], NKX2-3 [21], TGFβR2 [22] and CD302 [23]. However, no studies have been undertaken to generate an integrated molecular view of these neoplasms – the “interactome”. The relevance of such an analysis is that the delineation of the transcriptome, as a global measure, offers a complete overview of the cellular machinery at an RNA level – the cellular “toolbox”. This information provides the basis whereby network analysis can be utilized to identify specific interactive pathways associated with e.g., proliferation and metastasis rather than individual components. The establishment of the integrative pathways regulating the biological functions that constitute malignancy will likely have substantial translational applications.

Transcriptomic analysis can thus be utilized to provide a better understanding of tumor development as well as neoplasia. Such analyses have been demonstrated to be of considerable utility in other tumor types e.g., breast, particularly when translated to the clinical setting. Thus, considerable advance has occurred by upgrading histopathology, where gene-based analyses have allowed for the development of PCR-based arrays as well as custom-built chips to assess breast cancer classification [24–26], metastases [27] as well as predict therapeutic responsiveness [28]. Circulating tumor cells can readily be detected through PCR applications – such approaches appear to be more sensitive than current capture-based techniques – and may be more informative especially because multiple, biologically informative genes identified from RNA analyses can be assessed e.g., in non-small cell lung cancer [29], prostate cancer [30] or colon cancer [31]. Finally, a logical framework for the development of therapeutic targets can be generated through *in silico*-based reverse engineering of transcriptome data – this has previously been used to identify signaling pathways e.g., CREB targets [10] as well as master regulators – cardinal, potentially targetable genes that regulate nodes in pathways [32, 33].

Given the absence of any large-scale transcriptome study and the lack of analytical homogeneity between different NET transcriptome studies, we reanalyzed two publically available small intestinal NET microarray datasets [20, 21] (ArrayExpress: E-GEOD-6272/E-TABM-389). In order to identify genes that constitute the intestinal “NETwork”, we used a strategy that included stringent quality control techniques consistent with differential expression and validated network-based approaches [10, 34–36]. Thereafter, we undertook qPCR to corroborate transcript alterations in candidate targets in an independent collection of NETs. Finally, we screened public databases (e.g., [37]) and published literature (e.g., [38]) to focus on validated signaling pathways and critical transcription factors. This approach allowed us to confirm or reconsider known disruptions in signaling pathways in small intestinal NETs and identify pathways involved in development as well as novel transcription targets with putative therapeutic and biomarker potential.

## Results

### Sample set 1

**Figure 1:**
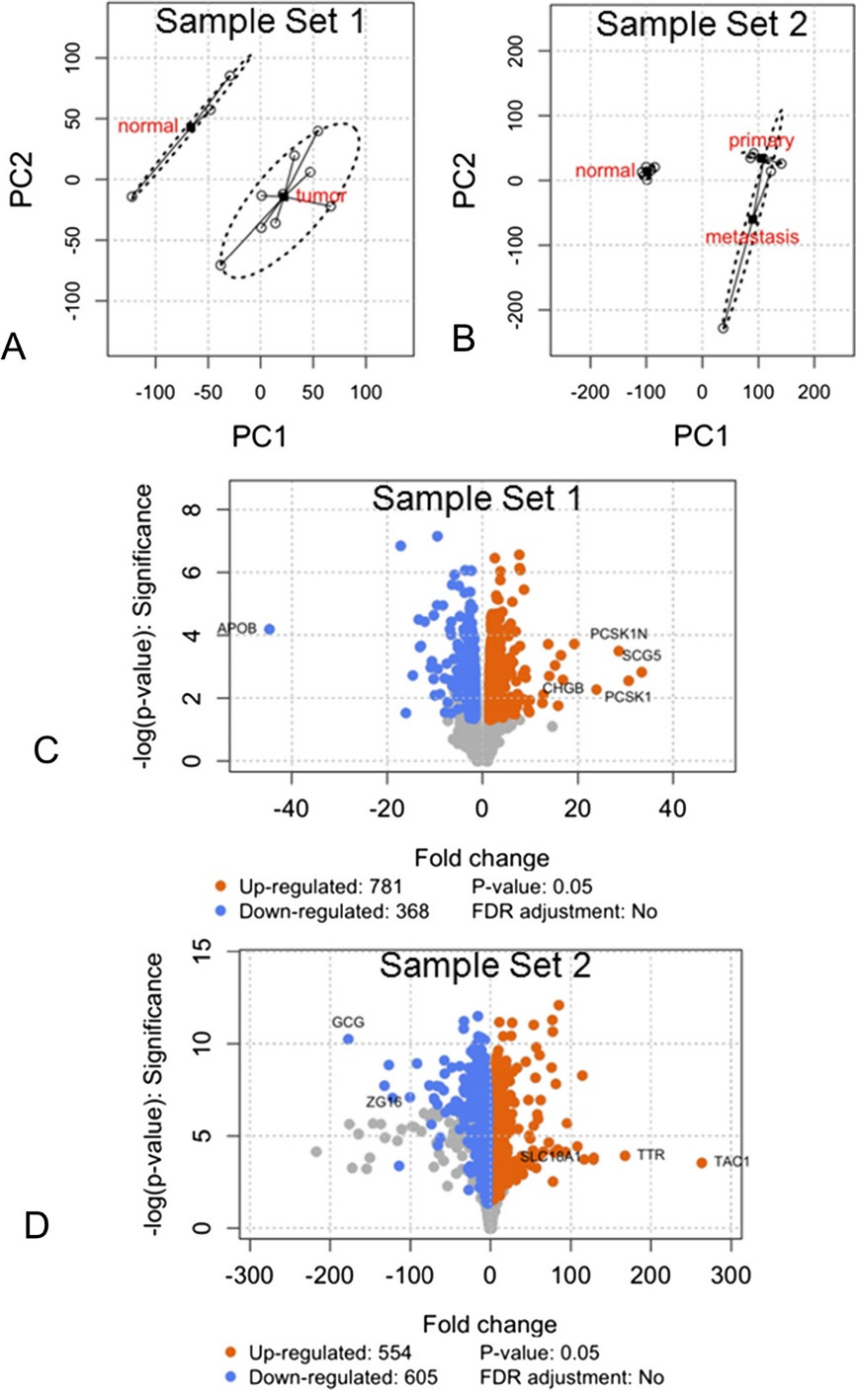
**Re-analysis of two small intestinal NET sets (** ***details in methodology*** **). A, B**. Principal component analysis and scatterplot of arrays along the first two principal components demonstrating spatial separation between control (normal mucosa) and tumor samples. **C, D**. Volcano plot of differentially expressed genes in Tumor compared to Normal for each of the sample sets. The most differentially expressed genes are labeled according to their fold changes.

**Figure 2:**
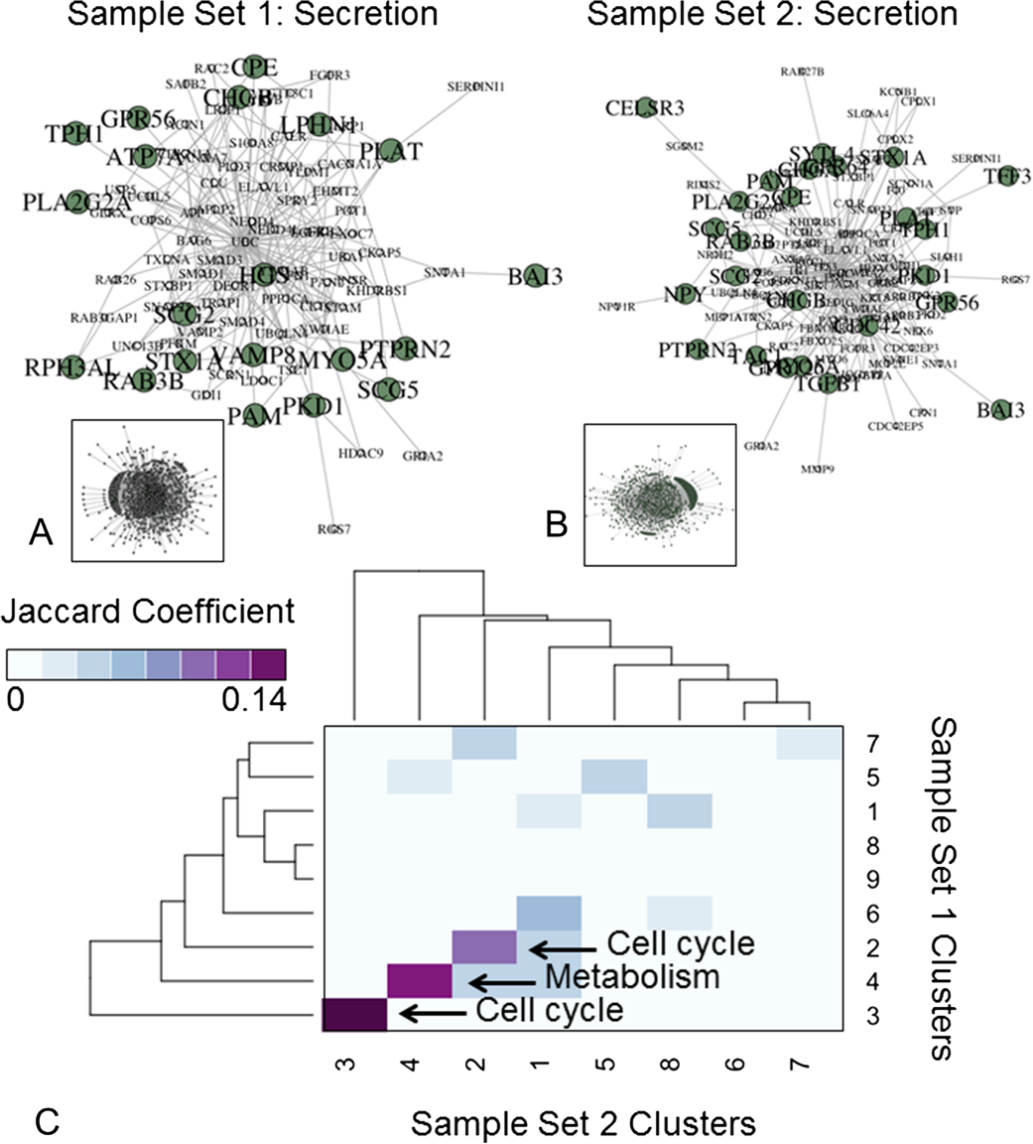
**Secretory interactome analysis of two small intestinal NET sets. A, B**. BioGRID secretory protein-protein interaction subnetworks of small intestinal NET microarrays. Proteins involved in secretory function are shown in green, while their neighbors are shown in white. Key genes in these pathways were examined by qPCR in the independent set (*see* Figures 3 and 4). **C**. Subnetwork cluster similarity heatmap. Darker shades reflect greater extent of shared proteins across network clusters in the two small intestinal NET protein-protein interaction subnetworks.

**Figure 3:**
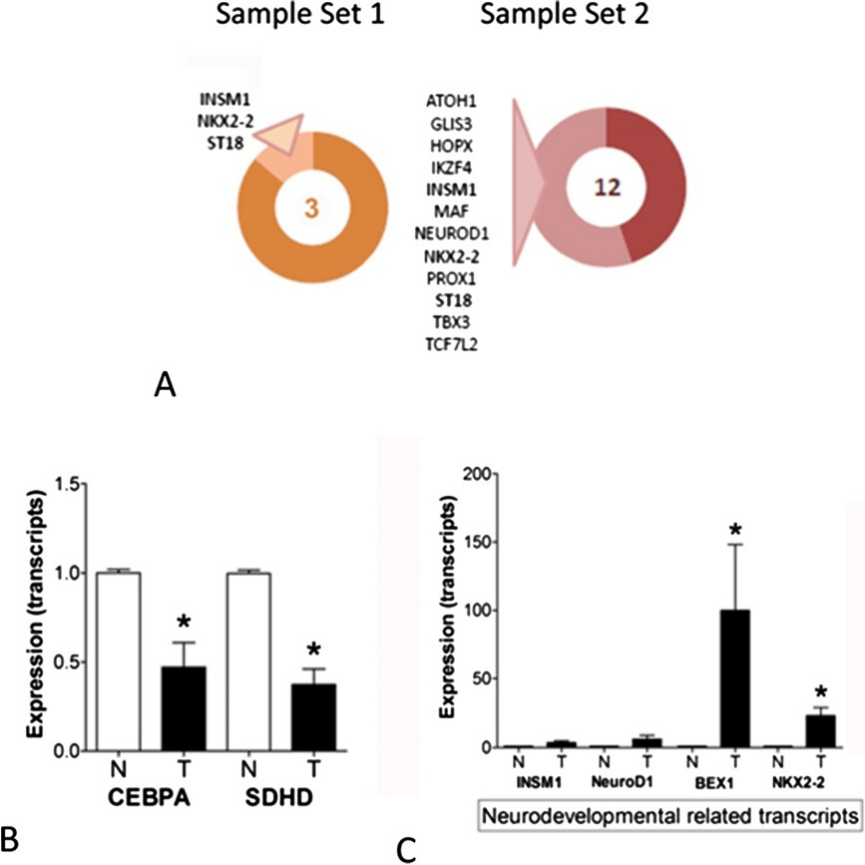
**Neurodevelopmental and COSMIC-based transcript expression in SI NET samples. A**. Enteroendocrine-related transcription factors in each of the data sets identified expression of 3 and 12 murine ortholog TFs, respectively. Commonly expressed TFs, involved in the regulation of neurodevelopment, included *INSM1*, *NKX2-2* and *ST18*. **B**. QPCR analysis of transcripts predicted by COSMIC analysis to be decreased in small intestinal NETs. Both *CEBPA* and *SDHD* expressed levels ~50% of normal mucosa consistent with a decreased expression and potentially a loss of function as has been noted in hematological cancers [71] and paragangliomas [39]. **C**. QPCR analysis of neurodevelopmental transcripts in the independent set confirmed elevated expression of *INSM1*, and *NEUROD1* and elevated expression of *BEX1* and *NKX2-2* validating the transcriptome-based analyses. Mean ± SEM, *p < 0.05 vs. normal mucosa. Tumors *n* = 13, normal mucosa *n* = 8.

Of the 22,283 features, 10,763 were present in more than 50% of total samples (*n* = 6) and therefore retained for further analysis. Overall, 7519 genes and 12 samples passed quality control procedures (see Additional file 1: Supplementary Methods, Additional file 2: Figure S1, Additional file 3: Figure S2 and Additional file 4: Figure S3) and were retained (Figure 1A, B). Of these, 781 up-regulated and 368 down-regulated genes were identified. The most differentially expressed genes are included in Table 1 and Figure 1C. Highly expressed genes included SCG5 (Fold change [FC] +33.4, *p* = 0.03), PCSK1 and PCSK1N (FC + 30.6-28.6, *p* < 0.05), SCN3A (FC + 19.2, *p* < 0.02), PNMA2 (FC + 16.3, *p* < 0.02) and NKX2-2 (FC + 15.2, *p* < 0.03). Additionally, differential expression analysis identified transcription factors such as INSM1 and NKX2-2, regulatory nucleoproteins including BEX1, PNMA2, AKT3, and CEBPA, transcripts involved in regulation of secretion through depolarization (e.g., SCN3A) and the regulation of insulin signaling and homeostasis (e.g., APLP1). Secretory protein subnetwork analysis identified members of the secretogranin family (e.g., SCG2, SCG3, SCG5) and involvement of the serotonin metabolic pathway (TPH1, ATP7A) (Figure 2A). Assessment of microarray expression of the 29 enteroendocrine transcription factors (TFs) previously identified in highly enriched gut endocrine cells [38], demonstrated the expression of four TFs including INSM1, NKX2-2 and ST18 (Figure 3A). Comparison of gene expression in Set 1 with the Sanger COSMIC dataset [37] identified five down-regulated genes that have previously been confirmed to result in neoplasia [39–43]; these included CEBPA, ERBB2, EXT1, PIM1, and SDHD. Differentially expressed genes and all functional enrichments are listed in Additional file 5: Table S1.

**Table 1:** **Highly elevated genes in each of the two sample sets based on microarray re-analysis**

Sample 1 [20]			Sample 2 [21]		
*Symbol*	*Fold change*	*Adjusted P-value*	*Symbol*	*Fold change*	*Adjusted P-value*
**SCG5**	+33.4	3.9E-02	**TAC1**	+263	1.6E-03
**PCSK1**	+30.7	5.2E-02	**TTR**	+167	8.5E-04
**PCSK1N**	+28.6	2E-02	**PCSK2**	+128	1.2E-03
**SCN3A**	+19.2	1.6E-02	**GPM6A**	+116	1.87E-06
**PNMA2**	+16.4	2.4E-02			
**NKX2-2**	+15.2	3.2E-02			

### Sample set 2

Of the 54,675 features, 12,420 genes passed quality control procedures and were retained. Differential expression analysis identified 554 up-regulated and 605 down-regulated genes. The most differentially expressed genes are shown in Table 1 and Figure 1D. Highly expressed genes included TAC1 (substance P/tachykinins: FC + 263, *p* < 10^-3^), TTR (FC + 167, *p* < 10^-4^) and PCSK2 (FC + 128, *p* < 10^-3^). Secretory protein subnetwork analysis identified a core set associated with secretion e.g., SCG2, SCG3, SCG5, SCN3A, serotonin metabolism (TPH1), and tachykinin receptor signaling (TAC1) (Figure 2B). Assessment of candidate enteroendocrine TFs identified expression of 12 TFs including INSM1, NEUROD1, NKX2-2, ST18 and TBX3 (Figure 3A). Comparison of gene expression in Set 2 with the Sanger COSMIC dataset identified twenty-nine down regulated genes previously confirmed to result in neoplasia; these included BCL11B, BUB1B, CANT1, CEBPA, EZR, FGFR2, HMGA1, HMGA2, LCK, MAF, MALT1, MYCL, POU2AF1, PPARG, PRDM1, and TNFRSF17. Differentially expressed genes and all functional enrichments are listed in Additional file 6: Table S2.

### Co-analysis of NET microarrays

**Figure 4:**
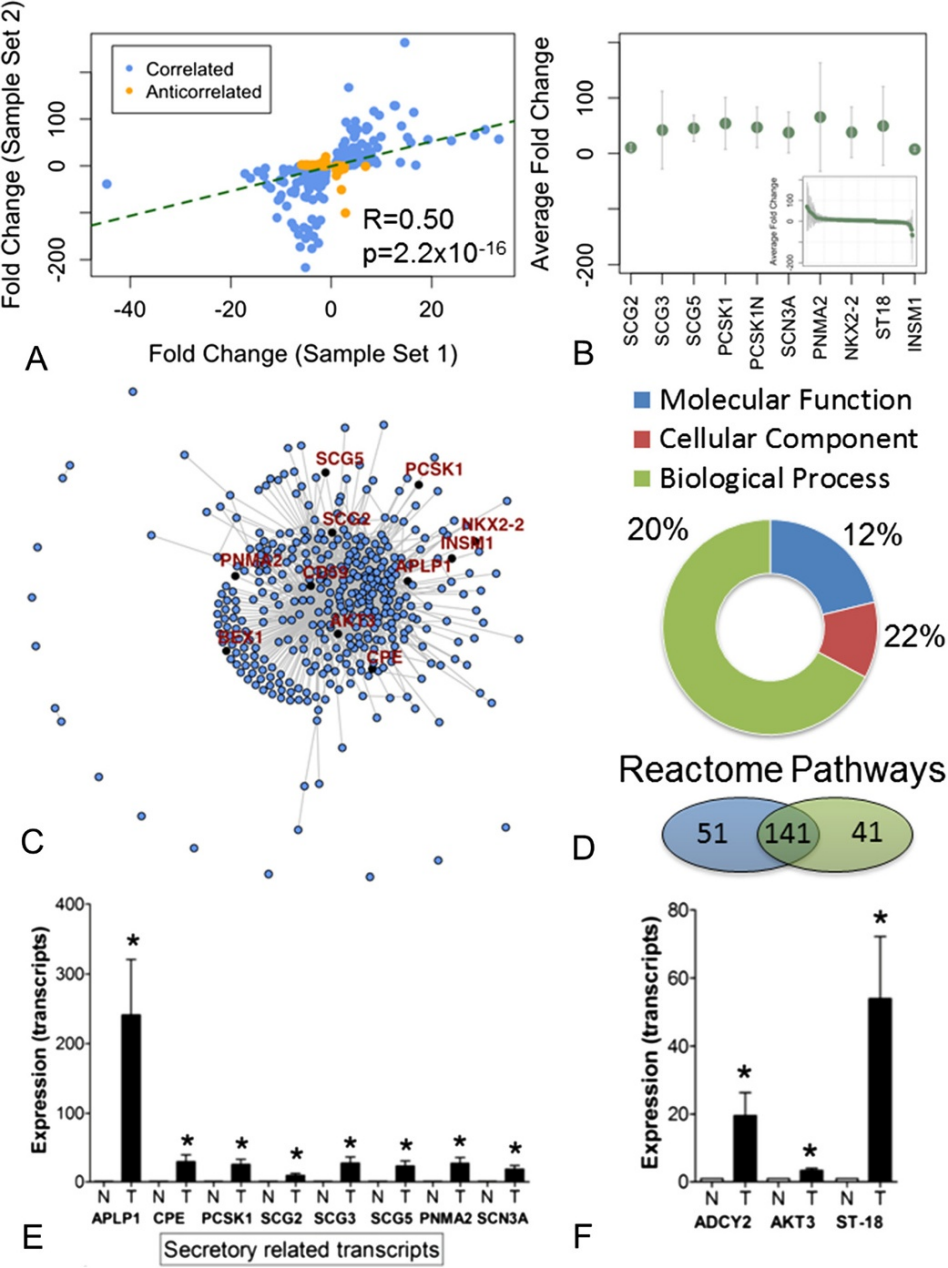
**Co-analyses of the two small intestinal NET sets. A**. Correlation profile of transcript alterations in each of the tumor sets. Both tissue databases were marginally correlated (R = 0.50). **B**. Commonly elevated transcripts in both datasets predominantly include genes involved in neuroendocrine secretion and regulation thereof. Error bars indicate the range of fold changes across the two datasets, while green points reflect average gene expression. **C**. Network analysis of the top ranked genes (*see* **B**) identified the most densely connected module to be related to secretion (interactome identified by multiple links). **D**. Gene-ontology and Reactome pathway demonstrating overlap between the two tumor sets; common pathways included secretion and xenobiotic responses (toxic environmental chemicals) as well as neurodevelopmental gene expression and alternative metabolic cycling (urea and TCA) consistent with a hypoxic phenotype (*see* Additional file 5: Table S1 and Additional file 6: Table S2). **E**. QPCR analysis of secretome-related transcripts in the independent set identified significant over-expression of all eight genes (ranging from *APLP1* to *SCN3A*). *p <0.05 vs. normal mucosa. **3F**. QPCR analysis of highly expressed transcripts in the independent set identified significant over-expression of *ADCY2*, *AKT3* and *ST18*. Mean ± SEM, *p < 0.05 vs. normal mucosa. Tumors *n* = 13, normal mucosa *n* = 8.

At the protein-protein interaction level, interactions involved in “Cell cycle” and “Metabolism” were the most conserved between the two datasets (Figure 2C). Additionally, a correlation was noted between changes in common gene expressions for Set 1 and Set 2 datasets (*n* = 7,299, R = 0.50, p = 2.2x10^-16^, Figure 4A). Interestingly, there were only 306 shared differentially expressed genes (26% of Set 1 and Set 2) between the two sample sets (Table 2). These included the SCG and PCSK family of genes, SCN3A, PNMA2, and the transcription factors, NKX2-2, ST18 and INSM1 (Figure 4B, C). At a Gene Ontology Biological Process level, the two tumor sets expressed overlapping enrichments in terms including “Secretion”, “Xenobiotic metabolic process”, and “Neuron development” (20% overlap) (Figure 4D). Similarly, overlapping Gene Ontology Cellular Component terms included “Secretory Granule” and “Vesicle Membrane” (22% overlap), while overlapping Molecular Process terms included “Voltage-gated Cation Channel Activity” and “Phospholipase Activity” (12% overlap) (Figure 4D). Reactome pathway analysis identified 73% overlap across significantly enriched pathways in Set 1 (*n* = 192) and Set 2 (*n* = 182); these included “Cell Cycle” and “Platelet Homeostasis (Figure 4D).

**Table 2:** **Commonly over-expressed genes in both datasets**

Concurrent analysis*		
***Symbol***	***Name***	***Process/function***
**SCG5**	Secretogranin V (7B2 protein)	Transport/Enzyme inhibitor activity
**PCSK1**	Proprotein convertase subtilisin/kexin type 1	Energy reserve metabolic process/Endopeptidase activity
**SCN3A**	Sodium channel, voltage-gated, type III, alpha subunit	Ion transport/Voltage-gated ion channel activity
**PNMA2**	Paraneoplastic Ma antigen 2	Apoptotic process/Protein binding
**NKX2-2**	NK2 homeobox 2	Type B pancreatic cell development/Core promoter proximal region DNA binding
**SCG2**	Secretogranin II	MAPK cascade/Cytokine activity
**ST18**	Suppression of tumorigenicity 18 (breast carcinoma) (zinc finger protein)	Negative regulation of transcription from RNA polymerase II promoter/DNA binding
**INSM1**	Insulinoma-associated 1	Regulation of transcription, DNA-dependent/DNA binding
**CPE**	Carboxypeptidase E	Cardiac left ventricle morphogenesis/Carboxypeptidase activity
**BEX1**	Brain expressed, X-linked 1	Multicellular organismal development/RNA polymerase II activating transcription factor binding
**APLP1**	Amyloid beta (A4) precursor-like protein 1	MRNA polyadenylation/Protein binding
**AKT3**	V-akt murine thymoma viral oncogene homolog 3 (protein kinase B, gamma)	Mitochondrial genome maintenance/Nucleotide binding
**CD59**	CD59 molecule, complement regulatory protein	Cell surface receptor signaling pathway/Protein binding

*This manuscript.

### PCR validation in independent set

qPCR analysis confirmed up regulated expression of 13/15 (87%) genes in small intestinal NETs compared to normal mucosa. Of the most expressed genes (identified at a transcriptome level), SCG5 (FC + 24, *p* < 0.04), PCSK1 (FC + 26, *p* <0.02), SCN3A (FC + 19, *p* <0.002), PNMA2 (FC + 27, *p* < 0.05), NKX2-2 (FC + 23, *p* <0.002), BEX1 (FC + 100, *p* < 0.002) and APLP1 (FC + 240, *p* = 0.01) were all highly expressed as was the transcription factor ST18 (FC + 43, *p* < 0.003) (Figure 4E-F). Transcripts associated with the COSMIC database and predicted to be down-regulated included SDHD (FC-2.5, *p* < 0.002) and CEBPA (FC-2, *p* < 0.02) (Figure 3B). Core regulatory genes involved in neurodevelopment were also expressed (FC + 3-6) (Figure 3C).

## Discussion

The precise basis of small intestinal tumor genomic profile has proven to be a complex subject and an integrated, cellular transcriptomic appreciation of neuroendocrine tumors has heretofore not been possible. This reflects a number of issues namely the paucity of studies available, the low number of tumor samples analyzed, the divergent analytical tools utilized and dissimilar focuses of the investigative groups e.g., focus on identifying metastatic genes [20]. We sought to define the issue using an integrated transcriptome analysis based on gene network-approaches that has successfully been proven to identify associations not previously apparent [10, 34–36]. Additionally, while it is likely that the current paradigm in tumor sequencing calls for tumor samples to be matched with control samples from the same individual [44], we hypothesized that comparing diverse population may shed light on tumor-specific behavior rather than on sample-specific behavior. Overall, the information derived (from two independent datasets) demonstrates four areas of novelty and considerable interest. Firstly, expression of core regulatory secretory regulatory elements, including genes involved in depolarization, was identified. The data therefore provide a complete overview of genes involved in regulated secretion and demonstrate the conservation of secretory apparatus in these tumors. Secondly, a set of transcription factors associated with neurodevelopmental processes including *INSM1*, *NKX2-2* and *BEX1* were identified indicating that the regulation of neuroendocrine differentiation occurs in tumors and that aberrations of this process may be of biological relevance in the evolution of the neoplastic phenotype. Thirdly, we confirmed loss of *SDHD* expression, a phenomenon associated with “benign” conditions in other tumors e.g., paragangliomas [39]. Finally, our data may suggest that at a genomic level small intestinal NETs may be distinguished by at least two distinct, secretory subtypes, serotonin-producing neoplasms and serotonin/substance P (TAC1/tachykinin)-producing lesions. As such, this is supported by previous studies in small intestinal NETs with “carcinoid syndrome” i.e., produce excess serotonin which suggests at least two subtypes of tumors. These include: 1) the demonstration that elevated luminal concentrations of substance P (secreted from mucosal sources) are only measured in 12% of patients [45]; 2) fasting circulating substance P concentrations are elevated in <20% of carcinoids [46]; and 3) at least two distinct serotonin producing NET lesions have been identified – serotonin producing NETs in the pancreas are TAC1/substance P negative [47].

### Serotonin-secreting tumors (Set 1)

Genome-wide co-expression analysis of these lesions [20] revealed processes including ‘Nervous system development’ (e.g., *BEX1*, *SYN1*, *GRIA2*), ‘Immune response’ (e.g., *CD38, IGKC, SLAMF8)*, and ‘Cell-cycle’ (e.g., *ASPM, MKI67, TOP2A)*. Importantly, gene network topology and differential expression analysis identified over-expression of the GPCR signaling regulators, cAMP synthetase (*ADCY2)*, and the protein kinase A, PRKAR1A. *ADCY2* was confirmed to be elevated in expression in our independent set; PRKAR1A and the role of cAMP-signaling have been previously studied in detail [10].

### Serotonin/substance P (TAC1)-secreting tumors (Set 2)

A reanalysis of the microarray data [21] identified over-expression of common genes with Set 1 including *APLP1*, *SCN3A*, *BEX*, *INSM1* and *ST18*. However, the most highly and uniquely expressed gene was *TAC1*, or substance P/tachykinins. Our secretory subnetwork analysis suggests that these tumors may not be classical serotonin-producing lesions.

### Combinatorial-analysis

This interactome assessment of the highly expressed genes identified canonical elements of secretory regulation including secretogranins, vesicle trafficking and hormone processing. The chromogranins (CgA and CgB), secretogranins (secretogranin II and secretogranin III), and additional related proteins e.g., PCSK1 and 2 (which are found within dense core secretory granules in endocrine and neuroendocrine cells and process several hormones and neuropeptide precursors), PNMA2 (a secreted protein that may generate autoantibodies [48]), APLP1 (which colocalizes with APLP2 and synaptophysin [49]), as well as carboxypeptidase E (CPE) have essential roles in the regulated secretory pathway or as products of this pathway [50]. Elevated expression of these genes was confirmed by qPCR in an independent set and provides evidence corroborating the secretome fingerprint of the tumor cells. Of interest was the identification of high expression of *SCN3A* (Nav1.3). This tetrodotoxin-sensitive voltage-gated sodium channel gene mediates membrane depolarization in excitable cells [51]. This suggests that this gene may be involved in regulating aspects of neuroendocrine secretion which mechanistically require a depolarization event. It is clinically well recognized that small intestinal tumors are sensitized to paroxysmal increased release of serotonin or substance P/tachykinins by secretagogues [52]. In this respect, Nav1.3 is increased in expression following nerve injury with the concomitant phenomenon of hyperalgesia in dorsal root ganglia [53]. We speculate that this elevated expression of Nav1.3 in neuroendocrine tumors may be related.

An assessment of the twenty-nine enteroendocrine-related transcription factors [38] identified that *ST18*, *INSM1* and *NKX2-2* were commonly expressed in both tumor sets. ST18 (Myt3) is a candidate tumor suppressor in breast cancer; ectopic expression in MCF-7 breast cancer cells strongly inhibits colony formation in soft agar and the formation of tumors in a xenograft mouse model [54]; it is also known to function as an pro-apoptotic effector [55]. This gene, however, is involved in neuronal differentiation [56] as well as in normal pancreatic islet cell development [57]. Interactome analysis of small intestinal NET transcriptomes identified neuroendocrine developmental pathways to be a key feature of these lesions. *INSM1*, *NKX2-2*, and *NEUROD1* were all identified to co-exist and elevated expression levels of these genes were confirmed by qPCR. Identification of other genes for example, TBX family members, in each transcriptome dataset supports a common activation of developmental pathways in these lesions and suggested the existence of a network of transactivating factors that function together to regulate the neuroendocrine phenotype. Further support for this is provided by over-expression of *BEX1* which is considered a regeneration-associated gene [58] and may be involved in tumorigenesis [59]. Bex1 is epigenetically activated in neurosphere cells and is considered relevant as a marker of reactivation of stem cell and pluripotency-associated genes; Bex1 expression enlarges the differentiation potential of precursor cells [60]. These data suggest that transcription factors that regulate neuroendocrine cell development or lineage specification are upregulated in neuroendocrine tumors as has been noted in lung tumors [61]. This may indicate an active control of the neuroendocrine phenotype in tumors but also raises the question as to whether an abnormal phenotype (i.e. less well-differentiated tumor) could occur as a consequence of a disruption in the TFs (e.g., through methylation-mediated repression) that co-ordinate the neurodevelopmental pathway. A similar phenomenon has been identified for tumor progenitor cells in small cell lung cancer [62].

At a developmental level, INSM1, apart from regulating neural and olfactory development [63], is essential for proper specification of both gastrointestinal and pancreatic endocrine cells [64] through interruption of cell cycle signaling, and cellular proliferation inhibition [65]. Endocrine transdifferentiation in BON cells is mediated by INSM1 through activation of NGN3 [66]. The plasticity of the neuroendocrine phenotype is controlled by NKX2-2 which regulates cell fate choices within the intestinal enteroendocrine population [67]. When this transcription factor is down-regulated, pancreatic alpha- and beta-cell development is impaired; the ghrelin-expressing cell population, in contrast, is augmented [68]. Upregulation of NKX2-2 is considered one of the primary regulatory events required for the maintenance of beta-cell identity [69]. Although the precise role of these genes in NETs is unclear, given the known roles in neuroendocrine development, it seems plausible that activation of neuroedevelopmental pathway (s) can be implicated in NET proliferation. INSM1, at least, functions through disruption of the cell cycle by targeting the CDK4/CyclinD1 complex.

A second gene linked to this complex is CEBPA (CCAAT/enhancer binding protein alpha (C/EBPalpha). This is a basic/leucine zipper transcription factor that integrates transcription with proliferation to regulate the differentiation of tissues involved in energy balance. In the pituitary, C/EBPalpha functions to prolong the cell cycle in G1 and S in pituitary progenitor cells [70]. An assessment of the 487 genes in the COSMIC database verified to be associated in a dominant or recessive fashion with cancer identified that *CEBPA* was down-regulated in both NET groups we studied. QPCR confirmed decreased expression of this gene (~50% of mucosal expression). Loss of function of this gene is associated with AML and MDS, largely through regulation of differentiation; this gene product inhibits CDK2/4 and the cyclin D1 pathway [71]. We postulate that a similar mechanism exists in small intestinal NETs; elevations in cdks and cyclin expression are well-recognized in NETs particularly as a consequence of IGF-1 stimulation [72]. It is noteworthy that inhibition of proliferation using interferons specifically inhibits these effectors *in vitro* [73].

A consistent loss or decrease in expression of *SDHD*, a recessive gene involved in paragangliomas, was noted in both tumor sets. Mutations in *SDHD* result in loss of complex II function and are associated with loss of stabilization of HIF1 under normoxia and generation of reactive oxygen species [74]. Mutations in this gene are considered to result in a “benign” phenotype in paraganglioma, the mechanisms of which are considered to be due to activation of cellular hypoxia responses [39]. Although no mutations have been detected in SDHD in intestinal NETs [75], LOH has been identified in ~30% of lesions [76]. Interestingly, LOH alone could lead to a complete loss of function since SDHD is an imprinted gene [39]. QPCR, in an independent dataset, confirmed decreased expression (~50% of normal mucosal levels) of *SDHD* indicating a potential role for hypoxia in intestinal tumor biology.

## Conclusions

We have identified two subtypes of intestinal neuroendocrine tumors, both associated with metastases, that express common signaling pathways involved in neuroendocrine secretion, nervous system and neuroendocrine development, as well as hypoxia and cyclin/CDK4 regulation. Transcriptome analyses have previously been leveraged to identify markers either of metastases [77] or blood-based antigens [48] or circulating transcripts [78]. The latter has evolved from a single transcript approach to a multiple gene screen – 51 marker genes – that are closely correlated with neuroendocrine tumor biology [79] and overlap with genes e.g., APLP1 family, PNMA2 and CD59, in the current study. Detection of this enhanced gene signature has been shown to be significantly more effective than measurements of chromogranin A by ELISA as a peripheral blood tool for detecting NETs [79]. In addition, because it is based on assessment of multiple NET transcriptomes it is also effective at identifying all gastroenteropancreatic lesions irrespective of the organ of origin and tumors including in the absence of metastasis.

This manuscript provides an integrated transcriptomic view of small intestinal neuroendocrine tumors and identifies that these lesions are regulated at a developmental level, have key activation of hypoxic pathways (a known regulator of malignant stem cell phenotypes) as well as activation of genes involved in apoptosis and proliferation. Further analyses and leverage of these data should provide novel tissue and blood-based tools to better understand, diagnose and ultimately treat these neoplasms.

## Methods

Please refer to the Additional file 1: Supplementary Methods for detailed description of computational protocols.

### Gene expression arrays and independent validation set

All samples were collected following informed consent and analyzed according to Ethics Committee requirements of Yale University (IRB: 0805003870; expires 6/18/2015) in accordance with the World Medical Association Declaration of Helsinki regarding ethical conduct of research involving human subjects [79]). Clinical details regarding the three samples sets are included in Table 3. No statistically significant differences were noted in distribution of gender, age or treatment received between each of the sets.

**Table 3:** **Demographics of NETs (Sample sets 1–3)**

Sample set	Sample no.	Gender	Age range	Site	Metastases	Treatment ^#^
1	T1	M	45-49	Ileum	N	N
1	T2	F	60-64	Ileum	N	N
1	T3	F	45-49	Ileum	N	N
1	T4	M	65-69	Ileum	N	N
1	T5	F	85-89	Ileum	N	N
1	T6	M	40-44	Ileum	N	N
1	T7	F	65-69	Ileum	N	N
1	T8	M	65-69	Ileum	N	N
1	T9	F	55-59	Ileum	N	N
2	T1	M	70-74	Ileum	N	N
2	T2	M	80-84	Ileocecal junction	N	N
2	T3	F	60-64	Ileum	N	N
2	T4	M	50-54	Liver*	Y	Y
2	T5	F	60-64	Liver*	Y	Y
2	T6	F	75-79	Liver*	Y	Y
3	T1	F	65-69	Ileum	N	N
3	T2	F	60-64	Ileum	Y	N
3	T3	M	65-69	Ileum	Y	Y
3	T4	M	65-69	Ileum	Y	Y
3	T5	F	60-64	Ileum	N	N
3	T6	M	75-79	Ileum	N	N
3	T7	F	60-64	Ileum	N	N
3	T8	F	55-59	Ileum	N	N
3	T9	M	40-44	Ileum	Y	N
3	T10	M	45-49	Ileum	N	N
3	T11	M	50-54	Ileum	N	N
3	T12	F	45-49	Ileum	N	N
3	T13	F	50-54	Ileum	Y	N

^#^Treatment included somatostatin analogs and/or interferon [21].

*All patients had carcinoid syndrome [21] so presumably the primary tumors were derived from the small intestine.

Female = female, M = Male, N = No, Y = Yes.

#### Sample set 1

Nine NET (obtained from the small intestine) transcriptomes and normal small intestinal mucosa (U133A chips, *n* = 9 tumors and *n* = 3 normal mucosa, ArrayExpress: E-GEOD-6272) [20]. Expression profiles were monitored across 22,283 probes.

#### Sample set 2

U133 Plus2 chips*, n* = 6 normal mucosa, *n* = 3 primary midgut NETs, and *n* = 3 GEP-NET metastases [METs] (ArrayExpress: E-TABM-389) [21].

#### Sample set 3 (Independent validation set)

Thirteen intestinal NETs (small intestine, including primary tumors: *n* = 8, liver metastases: *n* = 5) and eight normal small intestinal mucosa (matched samples) were collected. All samples were collected and analyzed according to a standard IRB protocol (Yale University: 6/5/2012) [79].

### Gene expression analyses

Individual analyses were performed using the web-based GeneProfiler tool (GeneProfiler, Bering Limited http://beringresearch.com/). Primary tumors were compared with non-matched normal mucosal samples. Sample set 1 consisted of 22,283 probes and 12 arrays, while sample set 2 consisted of 54,675 probes and 12 arrays. Probe sets that were unlikely to be reliable were eliminated using detection of Present/Absent calls. Probes present in more than 50% of samples were retained [80]. Raw probe intensities were normalized using the Robust Microarray Average (RMA) approach [81]. Array outlier detection was performed in the *arrayQualityMetrics* package [82] using the Kolmogorov-Smirnov statistic between each array’s distribution and the distribution of the pooled data. To enhance microarray annotation, probe identifiers (IDs) were mapped to Entrez Gene IDs (accessed April 7, 2013) [83]. In cases were multiple probes mapped to the same Entrez ID, the average probe intensity was calculated. Probes without an Entrez record were removed from analysis. Genes that were consistently identified as differentially expressed using multiple ranking algorithms [84] (fold change ranking, ordinary t-statistic, shrinkage t-statistic, limma, significance analysis of microarrays) were called significant and retained for further analysis. This approach ensured that differential expression analysis was: 1) unbiased, and 2) consistent across different array platforms.

### Functional gene expression analysis

Differentially expressed genes were enriched for Gene Ontology (GO) Biological Process (BP), Cellular Component (CC), and Molecular Function (MF) terms using the *topGO* Bioconductor package [85]. To ensure enrichment accuracy, terms with fewer than 10 assigned genes were not included in the analysis. Differentially expressed genes were also assessed at the Reactome pathway level (version 47) [86] using model-based gene set enrichment analysis [87].

For secondary analyses of selected genes, expression of genes relevant to carcinoma were assessed using the Sanger COSMIC database [37], while candidate enteroendocrine transcription factors were assessed against murine orthologs identified through transcriptome profiling of highly enriched populations [38]. The aim of these analyses was to assess the capacity to which differential expression analysis could identify previously known oncogenes and transcription factors.

### Protein-protein interaction network analysis

Differentially expressed genes (seed nodes) were mapped to human interactions obtained from the BioGRID database (version 3.2.109, *n* = 15,068 proteins and *n* = 124,370 interactions) [88]. High-scoring differential subnetworks were extracted and visualized to identify putative signaling regulators (see Additional file 1: Supplementary Methods, Additional file 2: Figure S1, Additional file 3: Figure S2 and Additional file 4: Figure S3 for a full description of the methods). Briefly, for each differential expression analysis, network nodes were assigned a weight of –log_10_(p-value). Subsequently, all shortest paths were calculated between seed nodes. Each shortest path was assigned a weight, expressed as the sum of nodes on that shortest path. A subnetwork was extracted by selecting seed nodes and “linker” nodes that fell on the highest weighted shortest path between the seed nodes.

Pairwise interaction network similarity was assessed by network community detection and subsequent calculation of inter-community similarity. For each network, protein communities were identified by optimizing the network modularity [89] (Additional file 1: Supplementary Methods**,** Additional file 2: Figure S1, Additional file 3: Figure S2 and Additional file 4: Figure S3). Similarity between protein communities was expressed using the Jaccard coefficient, computed as a ratio of the number of common proteins in any two network communities to the total number of proteins in these communities. Disparate and identical communities would correspond to Jaccard coefficient of 0 and 1 respectively.

Secretory protein subnetwork analyses were performed by extracting proteins from highly-scoring NET subnetworks involved in serotonin metabolism (GO:0042428, GO:0042427, GO:0007210, GO:0004993), substance P signaling (GO:0071861, GO:0007217), and secretion (GO:0007218, GO:0030141).

### Real-time PCR validation (Independent Set)

To validate candidate genes, we measured transcript expression in an independent Set 3 (SI NETs: *n* = 13, normal mucosa: *n* = 8) using real-time PCR. RNA was extracted (TRIZOL^®^, Invitrogen, USA) [90, 91] and real time RT-PCR analysis was performed using Assays-on-Demand™ products and the ABI 7900 Sequence Detection System according to the manufacturer’s suggestions [90, 91]. Primer probe sets are included in Table 4. Cycling was performed under standard conditions (TaqMan Universal PCR Master Mix Protocol) and data normalized (using *ALG9* and the ΔΔC_T_ method (Microsoft Excel). Non-parametric Mann–Whitney and Spearman correlations were used to compare samples and the Fisher’s test was used for binary comparison (GraphPad Prism 5).

**Table 4:** **Details of Applied Biosystems Primers (** ***n*** **= 18), including the housekeeping gene,** ***ALG9***

SI-NEN Biomarker or housekeeping gene		NCBI chromosome location	UniGene ID	RefSeq	Amplicon produced using forward and reverse primers	
Symbol	Name				Length	Exon boundary
ALG9*	Asparagine-linked glycosylation 9, alpha-1,2-mannosyltransferase homolog	Chr. 11–111652919 - 111742305	Hs.503850	NM_024740.2	68	4-5
ADCY2	Adenylate cyclase 2 (brain)	Chr.5: 7396343 - 7830194	Hs.481545	NM_020546.2	81	22-23
AKT3	v-akt murine thymoma viral oncogene homolog 3	Chr.1: 243651535 – 244006886	Hs.498292	NM_001206729.1	100	11-12
APLP1	Amyloid beta (A4) precursor-like protein 1	Chr.19: 36359401 – 36370699	Hs.74565	NM_001024807.1	142	11-12
BEX1	Brain expressed, X-linked 1	Chr.X: 102317581 – 102319168	Hs.334370	NM_018476.3	62	2-3
CEBPA	CCAAT/enhancer binding protein (C/EBP), alpha	Chr.19: 33790840 - 33793430	Hs.740432	NM_004364.3	77	1-1
CPE	carboxypeptidase E	Chr.4: 166300097 - 166419482	Hs.75360	NM_001873.2	106	7-8
INSM1	Insulinoma-associated 1	Chr.20: 20348765 - 20351593	Hs.89584	NM_002196.2	72	1-1
NEUROD1	Neuronal differentiation 1	Chr.2: 182541194 - 182545381	Hs.574626	NM_002500.4	110	2-2
NKX2-2	NK2 homeobox 2	Chr.20: 21491648 - 21494664	Hs.516922	NM_002509.3	114	1-2
PCSK1	Proprotein convertase subtilisin/kexin type 1	Chr.5: 95726040 - 95768985	Hs.78977	NM_000439.4	96	13-14
PNMA2	paraneoplastic Ma antigen 2	Chr.8: 26362196 - 26371483	Hs.591838	NM_007257.5	60	3-3
SCG2	Secretogranin II	Chr.2: 224461658 – 224467121	Hs.516726	NM_003469.4	69	1-2
SCG3	Secretogranin III	Chr.15: 51973550 - 52013223	Hs.232618	NM_001165257.1	92	5-6
SCG5	Secretogranin V	Chr.15: 32933870 - 32989298	s.156540	NM_001144757.1	84	5-6
SCN3A	Sodium channel, voltage-gated, type III, alpha subunit	Chr.2: 165944030 – 166060577	Hs.435274	NM_001081676.1	71	12-13
SDHD	Succinate dehydrogenase complex, subunit D, integral membrane protein	Chr.11: 111957571 – 111966518	Hs.356270	NM_003002.2	187	4-4
ST18	Suppression of tumorigenicity 18 (breast carcinoma) (zinc finger protein)	Chr.8: 53023392 – 53322439	Hs.655499	NM_014682.2	69	22-23

*ALG9 = housekeeping gene.

## Availability of supporting data section

Small intestinal neuroendocrine tumor microarray datasets are available from ArrayExpress:

### Dataset1

E-GEOD-6272 (http://www.ebi.ac.uk/arrayexpress/experiments/E-GEOD-6272/).

### Dataset2

E-TABM-389 (http://embl-ebi.org/arrayexpress/experiments/E-TABM-389/files/).

A supporting document with additional methodology information as well as 3 figures are included with this manuscript.

## Electronic supplementary material

Additional file 1: **Supplementary Information** [80–87, 92]**.** (DOCX 30 KB)

Additional file 2: Figure S1: GeneProfiler pipeline for microarray processing and quality control, differential expression analysis, and functional enrichment. (TIFF 214 KB)

Additional file 3: Figure S2: Overlap in the top 1000 differentially expressed genes between two datasets of the same tumor expressed as the Jaccard coefficient of similarity (number of genes in the intersection/number of genes in the union). (TIFF 158 KB)

Additional file 4: Figure S3: A toy graph to illustrate the implementation of our greatest-weighted shortest paths extraction algorithm. Seed nodes are shown in red, while linker nodes are shown in grey. The weight of each node is shown as a numerical label. (TIFF 70 KB)

Additional file 5: Table S1: Differentially expressed genes and functional enrichment of Sample Set 1. (XLS 582 KB)

Additional file 6: Table S2: Differentially expressed genes and functional enrichment of Sample Set 2. (XLS 642 KB)

## Abbreviations

CREB: cAMP Response Element Binding Protein
LOH: Loss of heterozygosity
NENs: Neuroendocrine neoplasms
NETs: Neuroendocrine tumors
QPCR: Quantitative PCR
SI: Small intestinal.
