# Supplementary material for: Gene network-based analysis identifies two potential subtypes of small intestinal neuroendocrine tumors
Source: BMC Genomics. 2014 Jul 15;15(1):595. doi: 10.1186/1471-2164-15-595 (PMC4124138; doi:10.1186/1471-2164-15-595)
Supplement: Supplementary file 1 — Additional file 1: Supplementary Information [80–87, 92]. (DOCX 30 KB) [file 12864_2014_6321_MOESM1_ESM.docx]

**Supplementary Information**

**Supplementary Methods & Results**

The microarray analysis pipeline is summarized in **Additional file 2: Figure S1**. This pipeline was applied individually to Sample Sets S1 and S2.

*Microarray processing and quality control*

Affymetrix CEL files were used as the GeneProfiler input. Raw probe sets that were unlikely to be reliable were eliminated using detection of Present/Absent calls. Probes present in more than 50% of samples were retained [80]. Raw probe intensities were normalized using the Robust Microarray Average (RMA) approach [81]. RMA consists of three steps: background adjustments, normalization, and summarization.

Array quality control was performed by detecting outlying microarrays. Detection was performed in the *arrayQualityMetrics* package [82] using the Kolmogorov-Smirnov statistic between each array's distribution and the distribution of the pooled data. To enhance microarray annotation, probe identifiers (IDs) were mapped to Entrez Gene IDs (accessed April 7, 2013) [83]. In cases were multiple probes mapped to the same Entrez ID, the average probe intensity was calculated. Probes without an Entrez record were removed from analysis.

*Differential expression analysis*

Usually, genes are filtered by virtue of their statistical significance, combined with a fold change threshold. These cut offs can be adjusted, depending on the distribution of differentially expressed genes. Such approaches have previously been shown to alter interpretations of microarray array studies [92].

To overcome this limitation, an automated significance threshold detection pipeline was used within GeneProfiler. In this approach, genes that were consistently identified as differentially expressed using multiple ranking algorithms [84] (fold change ranking, ordinary t-statistic, shrinkage t-statistic, limma, significance analysis of microarrays) were called significant and retained for further analysis. This approach ensures that differential expression analysis is: 1) unbiased and 2) consistent across different array platforms.

We further demonstrated reproducibility of automated differential expression profiling across studies performed in separate labs and on separate analysis platforms using a collection of multiple microarray datasets. Two independent datasets monitoring expression profiles in colon cancer (ArrayExpress: E-GEOD-44861, E-MTAB-57), pancreatic cancer (ArrayExpress: E-GEOD-28735, E-MEXP-950), and hepatocellular carcinoma (ArrayExpress: E-GEOD-6764, E-GEOD-14323) were assembled. Differential gene expression analysis was carried out for each dataset, comparing normal tissue with tumor tissue. We then measured the overlap in the top 1000 differentially expressed genes between two datasets of the same tumor using the Jaccard coefficient of similarity (number of genes in the intersection/number of genes in the union). If tumor profiles were different, similarity was expected to increase linearly as more and more genes were evaluated. Conversely, if tumor profiles were similar, an overlap was expected as new genes were assessed. Reproducibly similar differential expression profiles were seen in colon cancers, pancreatic cancers, and hepatocellular carcinoma. However, a linear increase in similarity was observed in the two NET datasets as well as in the two sets of random genes, suggesting that top differentially expressed genes in the two NET datasets are indeed different. (**Additional file 3: Figure S2**).

*Functional enrichment*

Differentially expressed genes were enriched for Gene Ontology (GO) Biological Process (BP), Cellular Component (CC), and Molecular Function (MF) terms using the *topGO* Bioconductor package [85]. The Classic Fischer test was used to test significance and p-values < 0.05 were considered statistically significant. To ensure enrichment accuracy, terms with fewer than 10 assigned genes were not included in the analysis. Differentially expressed genes were also assessed at the Reactome pathway level (version 47) [86] using model-based gene set enrichment analysis [87]. Terms with enrichment p-values < 0.05 were considered statistically significant.

*Protein-protein interaction network analysis*

Human protein-protein interactions were obtained from the BioGRID database (version 3.2.109, *n*=15,068 proteins and *n*=124,370 interactions). High-scoring differential subnetworks were extracted and visualized to identify putative signaling regulators. The procedure consists of the following steps and is highlighted in **Additional file 4: Figure S3**:

1. Map –log_10_(p-values) of all differentially expressed genes (seed nodes) to the protein-protein interaction network.
2. Calculate all shortest paths between all seed nodes.
3. Map –log_10_(p-values) of all genes to the shortest paths between seed nodes.
4. Calculate all shortest path weights by taking the sum of all –log_10_(p-values) of all the nodes along all shortest paths.
5. Retain only the highest weighted shortest path that connects any two seed nodes.

Shortest paths were calculated using the breadth-first search for unweighted graphs. In **Additional file 4: Figure S3** note that node *d* is selected to merge the seed nodes. Shortest paths between *d* and the seed nodes are used, generating a subgraph consisting of *b-d-c-e-g-f*. It is very important to note that node *a* and *d* have very similar topological positions in the graph, but their score differences drive the algorithm to select *d* eventually.
