# Supplementary figures and images for: Gene network-based analysis identifies two potential subtypes of small intestinal neuroendocrine tumors
Source: BMC Genomics. 2014 Jul 15;15(1):595. doi: 10.1186/1471-2164-15-595 (PMC4124138; doi:10.1186/1471-2164-15-595)

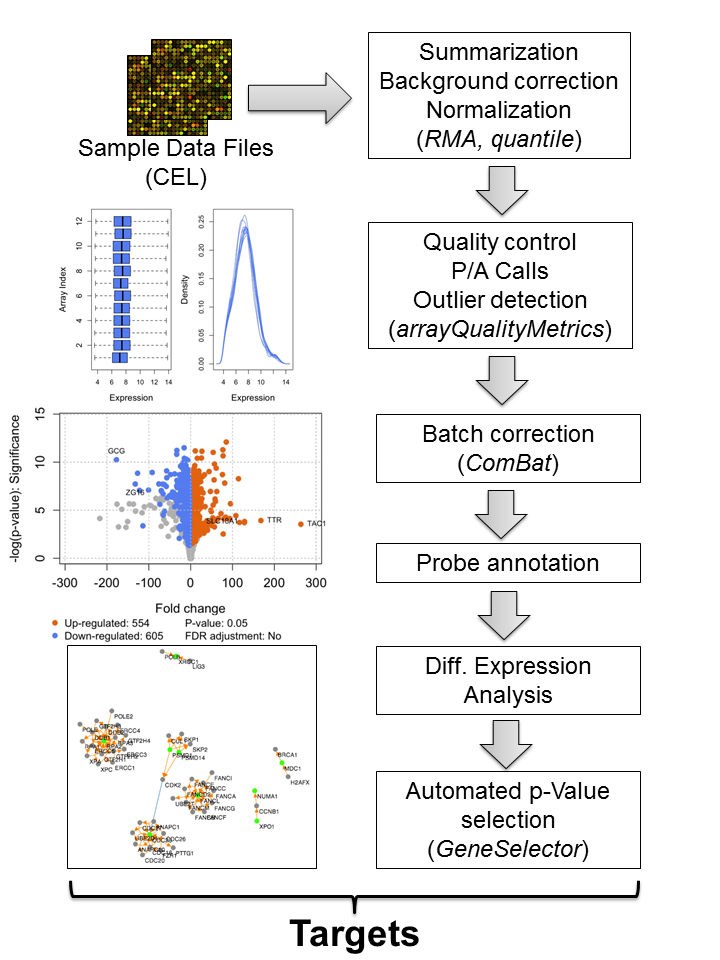

Supplement: Supplementary file 2 — Additional file 2: Figure S1: GeneProfiler pipeline for microarray processing and quality control, differential expression analysis, and functional enrichment. (TIFF 214 KB) [file 12864_2014_6321_MOESM2_ESM.tiff]

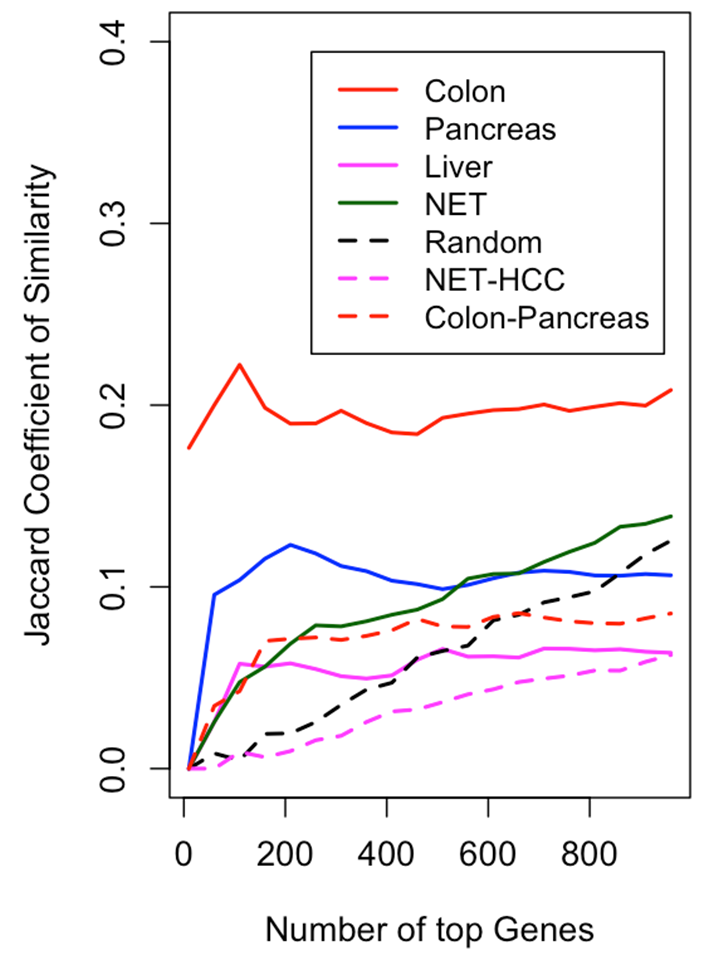

Supplement: Supplementary file 3 — Additional file 3: Figure S2: Overlap in the top 1000 differentially expressed genes between two datasets of the same tumor expressed as the Jaccard coefficient of similarity (number of genes in the intersection/number of genes in the union). (TIFF 158 KB) [file 12864_2014_6321_MOESM3_ESM.tiff]

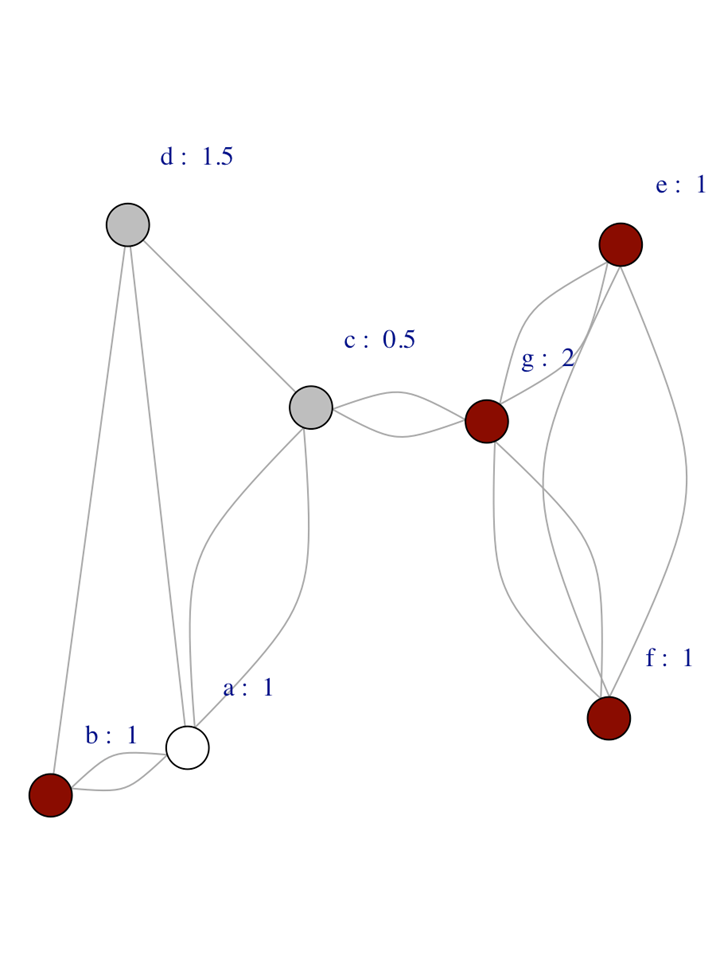

Supplement: Supplementary file 4 — Additional file 4: Figure S3: A toy graph to illustrate the implementation of our greatest-weighted shortest paths extraction algorithm. Seed nodes are shown in red, while linker nodes are shown in grey. The weight of each node is shown as a numerical label. (TIFF 70 KB) [file 12864_2014_6321_MOESM4_ESM.tiff]
